# Dysthymia through time: A review

**DOI:** 10.1192/j.eurpsy.2021.883

**Published:** 2021-08-13

**Authors:** M.T. Valadas, R. Mota Freitas

**Affiliations:** 1 Serviço De Psiquiatria, Unidade Local de Saúde do Baixo Alentejo, Beja, Portugal; 2 Departamento De Psiquiatria E Saúde Mental, Hospital do Espírito Santo de Évora, Évora, Portugal

**Keywords:** Dysthymia

## Abstract

**Introduction:**

Dysthymia is defined in ICD-10 as a chronic depression of mood which does not currently fulfil the criteria for recurrent depressive disorder, mild or moderate severity, in terms of either severity or duration of individual episodes. Although it only entered the psychiatric classifications in DSM-III and ICD-10, this syndrome has been a subject of several changes in conceptualization and classification.

**Objectives:**

We aim to perform an historical review on dysthymia and related concepts.

**Methods:**

We performed an updated review in the PubMed database using the terms “dysthymia”, “dysthymic disorder”, “persistent depressive disorder”, “neurotic depression” and “depressive personality”. The included articles were selected by title and abstract. We also consulted reference textbooks.

**Results:**

Depressive symptoms have been recognized since Antiquity, however, depressive disorders with a chronic course were only conceptualized in the 1970s. Dysthymia represents the confluence of older concepts, including neurotic depression and depressive personality and entered the psychiatric classifications in DSM-III and ICD-10. Presently, this syndrome is classified as persistent depressive disorder (dysthymia) in DSM-5 and named dysthymic disorder in ICD-11.

**Conclusions:**

The concepts regarding mental illness and psychiatric diagnoses are constantly evolving. Having knowledge about historical concepts is essential for a clear communication among psychiatrists, adding to the differential diagnosis process and improving patient care.

